# Role of MicroRNAs in the Development and Progression of the Four Medulloblastoma Subgroups

**DOI:** 10.3390/cancers13246323

**Published:** 2021-12-16

**Authors:** Emilia Bevacqua, Jasmin Farshchi, Maria Victoria Niklison-Chirou, Paola Tucci

**Affiliations:** 1 Department of Pharmacy, Health and Nutritional Sciences, University of Calabria, 87036 Rende, Italy; emilia.bevacqua@unical.it; 2 Centre for Therapeutic Innovation (CTI-Bath), Department of Pharmacy & Pharmacology, University of Bath, Bath BA2 7AY, UK; jkf33@bath.ac.uk

**Keywords:** miRNA, medulloblastoma, brain tumour, subgroups, stem cells

## Abstract

**Simple Summary:**

Medulloblastoma is the most common malignant paediatric brain tumour. Medulloblastoma originates in the cerebellum, a structure located at the base of the brain, affecting movement and balance in patients. Due to DNA alterations, known as mutation, some immature cells acquire new properties, transform from healthy cells into cancer cells and begin multiplying uncontrollably. During carcinogenesis, microRNAs (miRNAs or miRs) play important roles in medulloblastoma, helping cells to proliferate (oncomiRs) or inhibiting cell proliferation and promoting cell differentiation (tumour suppressor miRs). Therefore, in this review, we summarize the role of miRNAs in the four medulloblastoma subgroups and the importance of these non-coding RNAs to provide potential therapeutic applications.

**Abstract:**

Medulloblastoma is the most frequent malignant brain tumour in children. Medulloblastoma originate during the embryonic stage. They are located in the cerebellum, which is the area of the central nervous system (CNS) responsible for controlling equilibrium and coordination of movements. In 2012, medulloblastoma were divided into four subgroups based on a genome-wide analysis of RNA expression. These subgroups are named Wingless, Sonic Hedgehog, Group 3 and Group 4. Each subgroup has a different cell of origin, prognosis, and response to therapies. Wingless and Sonic Hedgehog medulloblastoma are so named based on the main mutation originating these tumours. Group 3 and Group 4 have generic names because we do not know the key mutation driving these tumours. Gene expression at the post-transcriptional level is regulated by a group of small single-stranded non-coding RNAs. These microRNA (miRNAs or miRs) play a central role in several cellular functions such as cell differentiation and, therefore, any malfunction in this regulatory system leads to a variety of disorders such as cancer. The role of miRNAs in medulloblastoma is still a topic of intense clinical research; previous studies have mostly concentrated on the clinical entity of the single disease rather than in the four molecular subgroups. In this review, we summarize the latest discoveries on miRNAs in the four medulloblastoma subgroups.

## 1. Introduction

Medulloblastoma (MB) is the most common primary malignant solid tumour of the central nervous system (CNS) in children and originates in a region of the brain known as cerebellum [1]. Embryonic tumours of the CNS account for approximately 4% of childhood cancers [2]. In Italy, according to AIRTUM (Italian Association of Cancer Registries) data, about 7 children per year out of a million are affected by this type of disease [3]. The incidence is slightly higher among males than females and is higher in younger children. Moreover, children with certain genetic diseases, such as Turcot syndrome, Gorlin syndrome, Li-Fraumeni syndrome, are at greater risk of developing medulloblastoma [4,5].

The symptoms related to a medulloblastoma depend on the tumour’s size and location [6]. The most common symptoms of medulloblastoma are headache, nausea and vomiting, progressive instability in walking, problems with coordination of the hands, arms, legs or feet, difficulty synchronizing eye movements, and changes in modulation of the voice [7].

Medulloblastoma is caused by different gene mutations, which can transform a healthy cell into a tumour cell [8]. It has been shown, following the discovery of miRNAs, that gene regulation can be altered at different levels, thus leading to tumour formation [9].

In 2005, it was reported for the first time that miRNAs play a central role in brain tumour development [10]. Since then, several studies have been performed in order to shed light on the role of miRNAs in brain tumours such as medulloblastoma in both paediatric and adult populations. Importantly, by using next-generation sequencing in a large cohort of medulloblastoma patients, common driver mutations have been revealed in each medulloblastoma subgroup [11]. However, the role of miRNAs in the framework of the different subgroups is still limited, since most of the studies have concentrated on the clinical entity of the single disease.

In this review, we highlight the major findings on the role of the miRNAs in the development and progression of medulloblastoma, their potential as biomarkers for cancer diagnosis, prognosis and therapeutic applications, with a particular focus on the regulation of the miRNAs in the four different medulloblastoma subgroups.

## 2. Medulloblastoma’s Classification

Medulloblastoma can be classified into different subgroups, which are distinguished based on how they present under the microscope (histological classification) or genetic alterations.

According to the World Health Organization (WHO), histological classification distinguishes four forms [12]:

- Classic, that is the most common subtype;
- Desmoplastic/nodular;
- Extensive nodularity, that is predominantly in infants;
- Anaplastic/large cell.

The classic forms, desmoplastic and with extensive nodularity, generally have a more favourable prognosis, while the anaplastic large cell form is the more aggressive [1] and displays high levels of atypia.

A more recent classification, based on genomics data, also divides medulloblastomas into four subgroups known as Wingless (WNT) and Sonic Hedgehog (SHH), which are better described and Group 3 (Grp3) and Group 4 (Grp4) less characterized. These new medulloblastoma entities are based on the presence of a specific gene mutation or amplification that causes cell proliferation [13,14].

### Diagnosis, Prognosis and Therapy

The diagnosis of medulloblastoma is made with imaging techniques such as computed tomography (CT) scan and, subsequently, magnetic resonance imaging (MRI) within one to three months from the appearance of the first symptoms, since medulloblastoma is a rapidly growing tumour [15]. Given the possibility of metastasis to other regions of the CNS, it is always essential to obtain images of both the brain and the spinal cord [13]. A cerebrospinal fluid sampling by lumbar puncture allows to exclude the presence of neoplastic cells at this level. Confirmation by histological examination is obtained after surgery to remove the tumour [16]. Dissemination outside the CNS is very rare.

The evolution of the disease (prognosis) and response to therapies are mainly linked to the medulloblastoma subgroup and to the presence of metastasis at diagnosis, although generally these tumours respond to therapies much better than other neoplasms of embryonic origin. The presence of metastasis in medulloblastoma is a poor prognostic factor. The treatment options for patients with metastases are limited. Unfortunately, it is not uncommon, even if the therapies have worked, for the tumour to reoccur after some time (relapse). In this case, the treatments are generally ineffective [17]. The 5-year survival from diagnosis is around 60–70% [6].

The therapy of choice for medulloblastoma is surgical removal of the tumour followed by chemotherapy and radiotherapy [18]. Ideally, the operation should completely remove all cancer cells, but it may be impractical if the tumour is in an inaccessible area or if there is a risk of damaging an area of vital importance or compromising the physical and cognitive functions of the patient [19]. Complementary therapy to surgery is direct radiotherapy to the head and spine (craniospinal radiotherapy) [20]. Over the decades, radiotherapy techniques and doses, both on the entire CNS and on the site of origin of the disease, have evolved and been modulated in order to make the treatment more effective and less harmful. The introduction of chemotherapy also contributed to this, which, depending on the initial situation, can be used after radiotherapy or before it. In special cases, in relation to the patient’s age, histological type and genetic subgroup, it is possible to reduce the total doses of radiotherapy or even omit it. It is important that the treatment plan also includes a rehabilitation path, which improves both the response to treatment and the quality of life of the young patient [21]. Finally, as in all paediatric diseases, an adequate and prolonged follow-up is essential in order to offer the best possible quality of life to the patient treated for cancer.

## 3. miRNAs

MiRNAs are small, non-coding regions in RNAs of around 22 nucleotides (nt) [22], that induce translational repression or degradation of a target mRNA upon imperfect base pairing to its 3′ untranslated region (3′UTR).

Initially, the biogenesis of miRNAs occurs in the nucleus with the transcription of the miR by an enzyme called RNA-polymerase II. The miRNAs derive from a primary precursor (pri-miRNA) of 100–1000 nt. The formation of mature miRNA occurs in three phases, the first still in the nucleus, the other two in the cytosol: (i) Cropping: cutting performed by RNAse III enzyme Drosha capable of cutting the region flanking the pri-miRNA. Other proteins that confer specificity are associated with the Drosha enzyme (ex. DGCR8). Following the cropping and the action of Drosha, the pre-miRNA composed of 80 nt is released, with a stem-loop structure, it has a 5′P and a 3′OH and 2–3 nt at the 3′OH end single helix protruding; (ii) Export: the pre-miRNA is transported into the cytoplasm by Exportin5/RanGTP, a heterodimer is formed which passes through the nuclear pores; (iii) Dicing: the pre-miRNA undergoes a further cleavage by another RNAse III enzyme Dicer which, together with its partner TRBP (HIV-1 TAR RNA RBP), process the pre-miRNA in a miR duplex of 18–22 nt. [23,24,25].

Then, while the mature miRNA duplex binds to AGO proteins forming RNA-induced silencing complex (RISC), in some cases, one of the two strands of the duplex is degraded, while the other accumulates as mature miRNA. From the duplex produced by Dicer, the miRNA enters in the protein effector complex RISC, with the presence of proteins belonging to the Argonauts family (AGO), which mediates the degradation or inhibition of mRNA translation of the target gene. In particular, the AGO2 protein, together with other proteins, forms the RISC multiprotein complex with endonuclease activity capable of specifically degrading a target RNA containing sequences complementary to the guide sequence of the miRNA. Eight members of the AGO family have been identified in the man. However, only the enzymatic function of the AGO2 protein is well known [26].

**Figure 1:**
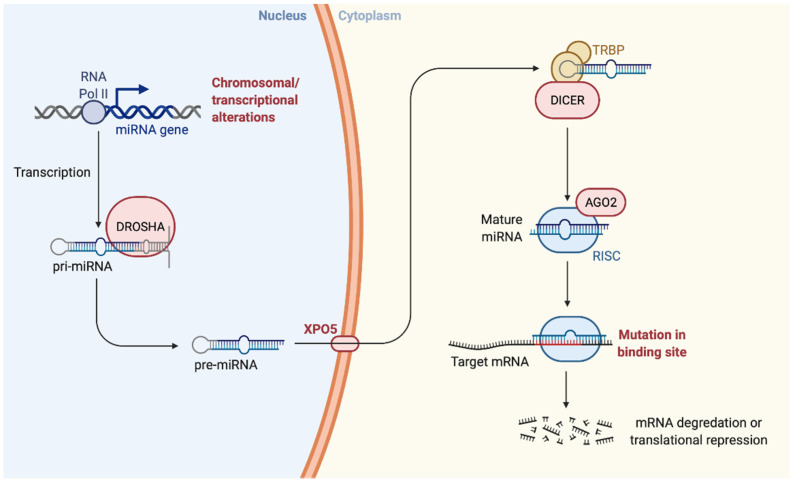
Biogenesis of the microRNA. “Created with BioRender.com”.

Some miRNAs appear imperfectly with the 3′UTR of the target mRNA and inhibit translation; other miRNAs show a precise complementarity to their target and lead to mRNA degradation. The biogenesis of miRNA and the mechanism by which they silence gene expression are represented in Figure 1.

MiRNAs are essential for the normal development of all tissues, as they control the most important biological processes such as cell growth, differentiation, metabolism and apoptosis [24]. For example, in Drosophila miR-14 prevents cell death and is required for normal lipid metabolism; miR-125b and miR let-7 control cell proliferation; miR-181 is involved in the development of the hematopoietic lineage of B lymphocytes [25]. MiR-15a and miR-16-1 promote the survival of immune B cells; miR-375 is involved in insulin secretion and miR-143 promotes the development of adipocytes [25,27].

### 3.1. Role of miRNAs in Neuronal Development

Nowadays, it is well accepted that miRNAs play a central role in several physiological processes. In particular, miRNA roles have been described during CNS development-related processes, response to ambient demands and injuries, stress or mental disorders. miRNAs are versatile regulators of gene expression, and they emerge as key players in numerous pathophysiological conditions, including CNS development, adaption and disease. Indeed, the significance of miRNA in development was confirmed by the fact that the loss of Dicer function causes lethal aberrations. It is estimated that over 60% of documented miRNAs are detected in the adult brain, and many of these change their expression as the embryonic brain develops and matures [28]. Recent data have also shown that miRNAs are expressed in the vertebrate nervous system and that their expression is modulated by synaptic activity, essential for learning and memory formation [27]. Altered morphology and neuronal development can result from errors in post-transcriptional processes that are closely regulated by miRNAs. Specific miRNAs are expressed in different compartments of the neural axis, and it has been hypothesized that miRNA pathways play a dominant role in inducing neuronal fate and synaptic plasticity [29]. Since early brain development and later synaptic plasticity are also regulated by miRNAs, it has been hypothesized that neurological disorders are influenced by their expression or alteration [30,31]. Neuronal differentiation, excitability and function are controlled by neuronal-specific miRNAs. For example, the transition from neuronal precursor to mature neurons is caused by the increase in miR-9 and miR-124 and therefore in the differentiation of embryonic stem cells. Scientists have displayed that miR-9 determines an inhibition of neurogenesis along the anterior-posterior axis [32], while miR-124 represses neuron-specific splicing patterns [33]. Neuronal differentiation and neurite growth, on the other hand, is modulated by miR-7 and miR-214 (as compared to miR-1,-16 and -133a) [27]. Neurodegenerative diseases such as Parkinson’s, Alzheimer’s or cancer also involve a reduction in the function of specific miRNAs [34,35].

### 3.2. Role of miRNAs in Cancer

The miRNAs are recognized to play a central role in development as well as in cell growth and proliferation, in differentiation, apoptosis, cell cycle, and metabolism controlling the expression levels of many genes [36]. Consequently, the alterations in the expression of these small RNAs play a key role in a wide variety of human diseases, including cancer. The first evidence of the involvement of a miRNA in cancer was demonstrated by Calin et al., in 2002 [37]. Since then, many studies have reported miRNA dysregulation in various human diseases [38]. About 50% of human miRNAs annotated are located in fragile sites of the genome associated with cancer and, moreover, they have been found differentially expressed between tumour cells and normal cells. Some miRNAs are downregulated while others are overexpressed in cancer, suggesting that miRNAs can act as tumour suppressor genes or oncogenes, respectively [39]. The epigenetic regulation of miRNAs, the hypomethylation of DNA, the increase in DNA methylation and the disruption of histone modification patterns in the miRNA locus, are greater than the genes that encode proteins. The miRNA genes can be silenced in some types of human tumours by aberrant hypermethylation of CpG Island that surrounds it, or is close to the miRNA of histone modification genes [40]. DNA hypermethylation in breast, lung and colon carcinomas was favoured by a decrease in the expression of miR-9-1 [41], miR-124a and miR-145-5p [42].

The aberrant expression of miRNA may be due to mutations in its sequence that cause a reduction in the expression of mature miRNA or an altered regulation of the target gene [43]. The activity of these small regulatory elements can also be altered by genomic rearrangements such as deletions or duplications of the genomic region in which the miRNA is located, or translocations that relocate the miRNA under the control of a new promoter.

**Figure 2:**
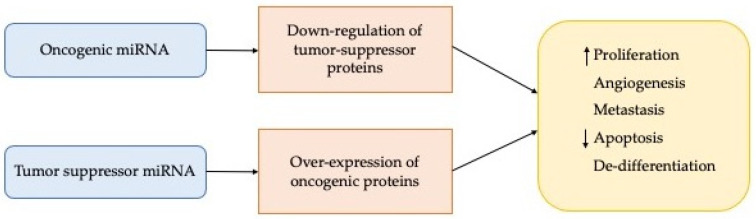
Main effects of deregulation of the miRNA expression.

The conclusive effects may be an increase in miRNA expression with a consequent decrease in expression of the target gene or a decrease in miR expression with a consequent overexpression of the target (Figure 2).

Despite the huge amount of miRNAs identified to date, their role in tumour processes is not entirely clear. However, the presence of miRNA circulating in the blood of cancer patients has increased the possibility that they could serve as new diagnostic and prognostic biomarkers, either alone or in combination with other well-stablished biomarkers [44].

In fact, some miRNAs are specifically more expressed only in one type of tumour, managing to also characterize malignancy [45,46]. Another reason for the choice of miRNAs as tumour biomarkers is to be found in the non-invasiveness of the analysis. In fact, miRNAs have been isolated from serum, plasma, saliva, urine and other cell fluids [46]. Several studies have shown that in these compartments the expression of miRNAs is correlated with specific tumours.

An early study [47] was concerned with identifying a tumour suppressor on chromosome 13q14, which was involved in chronic lymphocytic leukaemia (CLL), the most common form of leukaemia. They showed that the 13q14 locus does not contain genes encoding tumour suppressor proteins, but two microRNA genes, miR-15a and miR16-a, are expressed in the same polycistronic RNA. This result shows that the deletion of chromosome 13q14 would cause the loss of these two miRNAs, and therefore it is evident that these miRNAs are involved in the pathogenesis of human cancer.

Moreover, the discovery that miRNAs play a vital role in different types of tumour and since they have the advantage of being able to act both as oncogenes and as tumour suppressors they are still considered potential tumour therapeutic targets [48]. Carcinogenesis is favoured by oncogenic miRNAs, which are then over-expressed; on the contrary, the tumour suppressor action is due to a decrease in particular miRNAs (Figure 2). In light of the above, the antagomirs lead to a downregulation of the oncomirs. The concept of “miR replacement therapy” was thus introduced thanks to the observation of the reduction of the pathology following the action of suppressive miRNAs, with the aim of increasing the amount of reduced miRNAs and bringing them to normal values. This approach has great potential to be a more practical strategy than silencing individual genes by siRNAs and represents one of the major commercial areas of interest in today’s biotechnology market.

## 4. miRNAs Involvement in the Different Subgroups of Medulloblastoma

In 2012, it was agreed during an international meeting that medulloblastoma has four distinctive molecular subgroups named: Wingless (WNT-good prognosis), Sonic Hedgehog (SHH-intermedia prognosis), Group 3 (Grp3-bad prognosis) and Group 4 (Grp4-intermedia prognosis) [49]. WNT and SHH are named because these tumours have mutations in the WNT and SHH signalling pathways, respectively. To date, no clear underlying signalling pathways associated with Grp3 and Grp4 have been identified. Emerging evidence suggests that each group may require specific therapeutic strategies [50].

### 4.1. Wingless (WNT) Subgroup

WNT represent 10% of all medulloblastomas cases [51]. It occurs typically in adolescents and children over the age of 4 and is associated with excellent prognosis (>95% survival at 5 years in paediatric patients) [52]. WNT primary tumours are driven by a mutation in the CTNNB1 gene, which encodes b-catenin [53]. Mutation in this gene causes a constitutively upregulation of gene expression that promotes tumour growth and proliferation. Patients with WNT subgroups harbour TP53 mutations. In fact, WNT with p53 mutation have an excellent prognosis, suggesting that TP53-inactivating mutations on their own do not confer a poor survival [54]. Interestingly, the robust therapeutic response is attributed to an aberrant fenestrated vascular endothelium in the tumour. The fenestrated endothelial surface allows the accumulation of high levels of chemotherapeutic drugs in the tumour, thereby enhancing treatment [55]. However, children with a WNT diagnosis, are predisposed to primary tumour haemorrhage which can lead to severe complications [56]. Due to excellent prognosis of the WNT subgroup, a new clinical trial has been recently created to evaluate the reduction in chemotherapy and radiotherapy doses [57]. It was reported that miR-383, miR-206, miR-183, miR-128a/b and miR-133b are downregulated in this medulloblastoma subgroup [58] and the level expression of miR-449 is also completely different from other MB subtypes. miR-449 is down-regulated by aberrant DNA methylation in the WNT Group [59]. It was found that miR-148a expression is regulated by the NRP1 target. NRP1 target is involved in several pathways promoting tumour growth, invasion and metastasis. The downregulation of this target is due to the tumour suppressive effect of miR-148a expression and the subsequent reduction in tumorigenicity [60].

### 4.2. Sonic Hedgehog (SHH) Subgroup

SHH represents approximately 30% of MB cases and appears typically in infants and adults, accounting for two thirds of cases in these age groups [61]. The prognosis of this subgroup varies based on age and metastatic status and molecular mutations. It has recently been shown that p53, a tumour suppressor protein, is a prognostic marker for SHH-MB patients. In fact, patients with mutations of *TP53* gene have a worse outcome of the disease than those with wild-type *TP53* [62]. The altered SHH signalling pathway is mainly caused by germline or somatic mutation or copy number alterations in the SHH signalling pathway, which leads to tumour development and proliferation. The most common mutations are protein patched homolog (PTCH) inactivating mutation and smoothened homolog (SMO) activating mutation [63]. In fact, infant (35%) and children (45%) have mutations in the downstream SMO pathway, which makes tumours intrinsically resistant to SMO inhibitors [63]. Therefore, the recent approaches to modulate SHH signalling is focused on SMO inhibition and the mechanisms of acquired resistance in downstream SMO pathway. The metastasis of SHH subgroup happens at the same site of primary tumour. In a recent study, cancer stem cells (CSCs) have been isolated from SHH and expression level of epithelial to mesenchymal transition (EMT) transcript and microRNAs was compared with cerebellar NSCs [5]. Vegfa and its receptor Nrp2 are two molecules up regulated in SHH CSCs and involved in EMT [64]. If these two molecules are inhibited there will be a reduction of the cell viability and the ability of CSCs to self-renew. This mechanism leads to the modulation of two markers involved in EMT, therefore we will see the increase in the epithelial marker (E-Cadherin) and, on the other hand, a reduction of the mesenchymal one (Vimentin). The miRNA identified as an inhibitor of Vegfa and Nrp2 is miR-466-3p [64].

Furthermore, CSCs identified in SHH-MB are controlled by the Sonic Hedgehog/Gli (Hh/Gli) is an aberrant signalling pathway that control CSCs identified in SHH medulloblastoma, regulated by miR-326. More precisely, the downregulation of miR-326 is characteristic of these tumours, therefore an overexpression of miR-326 leads to the inhibition of that signalling pathway [65].

More recently, in vivo and in vitro studies have displayed that SHH MB cells showed a reduction in tumour growth by silencing miR-17/20 and miR-19a/b [66]. Furthermore, miR-17-92 cluster is involved in SHH tumours. Within this cluster belong miR-18a, -19a, -20a, -21, -25 and -106b [67]. Several studies have evaluated the effect of miR-10b on the growth and proliferation of medulloblastoma through the transcriptional induction of BCL-2, a tumour promoter [68]. Potent inhibitors of BCL-2, such as ABT-737 and ABT-199, were evaluated on the expression of miR-10b [69]. Powerful BCL-2 inhibitors significantly inhibit the expression of miR-10b in a dose-dependent manner. This miRNA is strongly associated with tumours, as it plays a crucial role in cell proliferation and survival, moreover miR-10b is not expressed in a normal brain. Several studies suggest that miR-10b is an oncomiR that regulates cell growth and survival of this medulloblastoma subgroup by controlling BCL2 levels [68].

### 4.3. Group 3 (Grp3) Subgroup

Grp3-MB is the most aggressive paediatric brain tumour and occurs mostly in infants and young children. This subgroup is (40–45%) metastasis at diagnosis and is resistant to combinations of surgery, radiotherapy and chemotherapy [53]. Therefore, it is associated with poor prognosis and the worst survival outcome of any subgroup (under 60% at 5 years). Unlike WNT and SHH subgroups, there is no distinctive altered signalling pathway identified for Grp3. However, amplification of MYC (17%) and hyper activation of the GFI1B oncogene (15–20%) are mostly observed. TP53 mutations were almost never observed in patients with Grp3, in which isochromosome 17q is a common aberration [54]. It was showed that miR-183-96-182 cluster are up-regulated in Group 3 of medulloblastoma. In particular, the expression of the miR-183 cluster in cells was associated with the dysfunction of the DNA damage repair and with the pathways associated with migration, EMT and metastasis [70]. Metastasis of Grp3 happens at a different site of the primary tumour. It was reported that, in medulloblastoma cell lines DAOY, D425 and D283 belonging, respectively, to the SHH subgroup and Grp3, there is an under-expression of miR-30a family [71]. Group 3 and 4 are associated with the highest mortality compared to other MB subgroups. Group 3 of MB displayed a deregulation of miR-1253 expression [72]. They showed that the restoration of miR-1253 expression is linked with a reduction in tumour cell malignancy, which also leads to the activation of apoptotic pathways. A recent study highlighted the expression of seven miRNAs belonging to the miR-30 family in the 4 subtypes of medulloblastoma. These miRNAs are significantly downregulated (*p* < 0.00001) compared to normal cerebellar tissues [71]. The inhibition of the clonogenic potential, proliferation and tumorigenicity of different cell lines of medulloblastoma is notable after the recovery of miR-30a through the lentiviral vector. MiR-30a is known to mediate autophagy through the Beclin1 target. Therefore, it has been shown that the expression of miR-30a leads to a down-regulation of genes implicated in autophagy, such as Beclin1, with consequent inhibition in medulloblastoma cells. Autophagy is a process that allows our cells to recycle and renew themselves. The cells then destroy their components that have become useless and carry them out of the membrane, playing a fundamental role in our defences. This process leads, on the one hand, to cleaning the cell, on the other hand it allows the cell to sustain itself in difficult situations [73]. Additionally, in the medulloblastoma, low levels of miR-4521 leads to an up-regulation of the transcription factor forkhead box M1 (FOXM1). FOXM1 regulates the expression of various genes involved in tumour progression [74]. The Grp3 is the only one with a statistically significant difference in miR-4521 expression reduction compared with the healthy control tissue, while in SHH subgroup there are no particular differences.

### 4.4. Group 4 (Grp4) Subgroup

Grp4 accounts for 35–40% of all medulloblastoma diagnosis and occurs typically in children and adolescence [75]. This subgroup is (35–40%) metastasis at diagnosis, although the survival outcomes are intermediate, and the recurrences mostly occur late. Grp4 share similar gen amplifications as Grp3 as mentioned above and have not an identified signalling pathway [76]. At the same time, Orthodenticle homeobox 2 (OXT2) amplification and the gain of isochromosome 17q is also seen in Grp4 and Grp3 [77]. Similar to Grp3, TP53 mutations has never been observed in Grp4 and the metastasis is at a different site of the primary tumour. Compared to the other subgroups, Group 4 has a lower expression of miR-181a-2-3p, which is reported to be involved in the formation of glioma acting as tumour suppressors [78,79]. While a high expression was observed for miR-187-3p and could be linked to a poor prognosis of patients with Group 4 MB [80]. Additionally, miR-206 was down-regulated in all four medulloblastoma subgroups. Indeed, miR-206 acts on OTX2, an oncogene which is involved in Grp4 pathogenesis. Overexpression of OTX2 leads to growth and proliferation of medulloblastoma. Therefore, under-expression of miR-206 contributed to the upregulation of OTX2 expression and enhanced growth of G4 cell lines [58]. A recent study showed that the tumour-suppressive let-7 miRNA family is downregulated by gene LIN28B and the expression of these miRNAs is significantly lower in Group 3 and 4 compared with WNT and SHH MB [81]. miR-4521 is located on chromosome 17p13.1. They show that a loss of chromosome 17p is closely associated with Grp3 and Grp4-subgroups [74].

## 5. Role of miRNAs in Medulloblastoma

The most studied tumours at the level of miRNA deregulation are breast, prostate, colon and leukaemia’s; little has been studied regarding the alterations of miRNAs in medulloblastoma. Ferretti et al. conducted one of the first studies on the expression profile of miRNAs in medulloblastoma [82]. A total of 250 miRNAs were screened in 31 primary medulloblastoma specimens and 34 miRNAs differentially expressed between SHH-MB versus WNT-MB, Grp3-MB and Grp4-MB were identified. Additionally, three down-regulated miRNAs were identified in SHH-MB, miR-125b, miR-326 and miR-324-5p. Interestingly, these three miRNAs are known to target Smoothened (SMO), an activator of the Hedgehog signalling pathway [82]. In addition, miR-324-5p also targets Gli1, a committed transcription factor for the Hedgehog signalling pathway. Additionally, it was suggested that a possible genetic anomaly is the cause of the loss of function of miRNA-324-5p in SHH-MB [83]. In fact, this miRNA is contained in a gene region of chromosome 17p, which is one of the most frequent deletions in medulloblastoma. In addition to the miRNA, this chromosomal region also contains the tumour suppressors p53 and HIC1 but also the antagonist of the REN signal pathway [84].

In a different study conducted by Ferretti et al., 248 miRNAs were analysed in medulloblastoma samples, and 248 miRNAs differentially expressed between tumours and normal adult and foetal cerebellar tissue were detected [85]. In this analysis, two miRNAs, miR-9 and miR-125a, were identified as downregulated in medulloblastoma and target the truncated isoform of the neurotropin receptor (TrkC). By comparing medulloblastoma and normal foetal cerebellum it was possible to identify a cluster of upregulated miRNAs in SHH-MB versus non-SHH medulloblastomas known as cluster 17-92 [67]. This miRNA cluster is induced by N-myc in the neuronal cerebellar precursors treated with Sonic Hedgehog; this evidence indicates that the 17-92 miRNAs cluster is a positive effector of the proliferative effects of the Hedgehog signalling pathway.

Additionally, Uziel et al. [86], using medulloblastoma cells from Ink4c ^−/−^; Ptch1 ^+/−^ and Ink4c ^−/−^; p53 ^−/−^ genetically modified mice versus mature mice, identified many deregulated miRNAs: 26 upregulated and 24 downregulated. In particular, 9 of these 26 upregulated miRNAs were demonstrated to encode cluster 17-92. To this cluster belong miR-92, miR-19a and miR-20 that are upregulated in the Hedgehog subgroup of medulloblastoma. Thus, demonstrating the close correlation between cluster 17-92 and the Hedgehog signalling pathway [86].

Two miRNAs (miR-30b and miR-30d) were identified located in a commonly amplified region in medulloblastoma, adjacent to the MYC locus on chromosome 8q24. Such miRNAs were found upregulated in a subgroup of primary medulloblastoma [87].

Cluster 183-96-182 was found upregulated in controlled non-Hedgehog medulloblastoma and, in particular, miR-182 was significantly upregulated in metastatic medulloblastoma [88]. It was later shown that this cluster is involved in the suppression of genes associated with apoptosis and the regulation of the PI3K/AKT/mTOR axis [89].

Venkataraman et al. showed that several miRNAs that are downregulated in medulloblastoma have an active role in normal brain development. In particular, miR-128a has been shown to be an antagonist of the Bmi1 oncogene [90].

Many miRNAs can influence tumorigenesis through their tumour suppressor action, such as miR-34a which if overexpressed in medulloblastoma cells and induces apoptosis and restores sensitivity to chemotherapy [89], or miR-199-5p that by its target HES1 regulate the cancer stem cells [91].

Therefore, miRNAs can potentially regulate several pathways involved in the insurgence and progression of the medulloblastoma, acting as both oncogene and tumour suppressor (summarized in Table 1).

### Clinical Application of miRNAs in Medulloblastoma

The epigenetic landscape, as well as DNA mutation or miRNAs expression of medulloblastoma, has been investigated for the last 20 years to discover novel biomarkers for diagnosis, treatment, and/or disease progression [122]. miRNAs analysis in medulloblastoma tissue samples, as well as in cerebrospinal fluid (CSF) and in blood has been performed [14,123]. Additionally, miRNA expression in extracellular vesicles isolated from CSF or blood has been investigated. Several miRNAs were found differentially expressed between the different MBs subgroups. Gershanov et al. [76], found three miRNAs differentially express in G4-MB. These miRNAs are miR-20a-5p, 181a-2-3p, and 224-5p. Additionally, Li et al. [59] reported that miR-449a is a very good candidate for WNT-MB. However, Yogi et al. [60] reported that miR-148a is a good candidate for WNT-MBs classification. However, due to the significant variation between samples (primary cells, cell lines, patients) and miRNA expression in these studies is making very difficult to select a miRNA or set of miRNAs to improve medulloblastoma diagnosis and treatment. Fortunately, with the onset of new techniques based on the study of miRNAs and the analysis of patients’ samples with medulloblastoma, miRNAs could be drastically improved to select aggressive versus non-aggressive medulloblastoma subgroups for treatment selection. Thus, considering their important roles in medulloblastoma development, miRNAs have been investigated as prognostic and diagnostic biomarkers for cancer detection, and also as useful targets for therapeutic intervention [124]. miRNA-based therapeutic treatments for medulloblastoma may follow the same strategies described above: miRNA over-expression or miRNA repression. However, the use of miRNAs as potential therapeutic targets for medulloblastoma remains controversial with regard to the ability of the miRNA delivery to pass through the blood–brain barrier. In order to overcome this limit, different systems to transport siRNA into the brain have been developed, such as engineered nanoparticles, vector-based, chemically modified, and “packaged” RNA oligonucleotides [95].

## 6. Conclusions

Medulloblastoma is a tumour of the paediatric population, the second most widespread brain tumour, after astrocytomas, and represents 1% of all cancers of the CNS [6]. A total of 70% of medulloblastomas are diagnosed between the second and tenth year of life. Survival five years from diagnosis (children and adults) is just over 60–70% [125].

Aberrant mechanisms of neuronal and cerebellar development can lead to the formation of medulloblastoma. These genetic and epigenetic changes can cause the abnormal activation of the Hedgehog signal pathway. In recent years, a hierarchical model for the evolution of cancer has been proposed, in which cancer stem cells (CSCs) acquire or maintain the properties of self-renewal, multipotency and tumour generation. This model has also found application in medulloblastoma, as CSCs have been observed in both mice and humans [126]. Furthermore, it was possible to demonstrate the correlation between the Hedgehog signalling pathway and these tumour cells [126], whose presence can lead to greater resistance to classical therapies and probability of relapse.

Most children die within three years due to aggressive treatment or recurrency [27]. Survivors must cope with severe long-term side effects; radiation of the entire developing brain and spinal cord to prevent metastatic recurrence have a devastating effect on intelligence, neurological and endocrine function [27]. Therefore, it is crucial to identify novel and effective therapeutic targets to treat these tumours and improve the quality of life of patients [127].

MiRNAs are known to play vital roles in nervous system development, as well as in various aspects of cancer development, progression, and metastasis. Thus, their involvement in medulloblastoma tumours is not surprising. In this review, we summarized the most important findings present in the literature on the role of miRNAs in influencing the tumorigenesis of medulloblastoma, inducing apoptosis and restoring sensitivity to chemotherapy [89,91]. Such as miR-326 that is absent in brain tumour pathologies and is involved in the modulation of signalling pathways, such as Hedgehog and Notch [82,85]. In particular, it interacts with the Hedgehog signalling pathway by negatively modulating the expression of the SMO activating receptor in cerebellar granules [82].

Furthermore, as we have seen, miRNAs are able not only to distinguish normal tissue from tumour, but also to characterize the different subgroups of medulloblastoma. Therefore, they can be used as biomarkers of tumour early diagnosis, prognosis, and provide new opportunities to treat the different clinical and biological features between subgroups.

In conclusion, it is crucial to know the functional and disease-associated mechanisms causing the deregulation of these small RNAs in medulloblastoma. Even though substantial questions must be answered, such as the role of the miRNAs in the development and progression of the different tumoral subgroups, they still represent a suitable target for the future medical treatment of medulloblastoma therapy, able to change the medical practice in the foreseeable future.

## Figures and Tables

**Table 1:** Summary of deregulated miRNAs involved in the pathogenesis and progression of the four MB subgroups.

SUBGROUP 1: WINGLESS					
miRNAs as Oncogenes	Cellular Function	Ref.	miRNAs as Suppressors	Cellular Function	Ref.
miR 30b, miR-30d	N/A	[87]	miR-9	Antiproliferation Differentiation Pro-apoptosis	[85,92]
miR-193a	Metastasis Proliferation	[93]	miR-148a	Antiproliferation Invasion Reduces tumorigenicity	[60,85,93]
miR-224	Proliferation Radiation-sensitivity. Anchorage-independent growth	[93,94]			
**SUBGROUP 2: SONIC HEDGEHOG**					
**miRNAs** **as Oncogenes**	**Cellular Function**	**Ref.**	**miRNAs** **as Suppressors**	**Cellular Function**	**Ref.**
miR-17/92	N-Myc target	[66,67,89]	miR-let-7	Chemoresistance	[82,85,95]
miR-183/96/182	Migration	[70]	miR-34a	Antiproliferation Pro-apoptosis Senescence	[89,96,97,98,99]
miR-196b-5p, miR-200b-3p	C-Myc target Proliferation Migration Invasion	[100]	miR-125b	Suppressing progenitor and tumor cell growth	[82]
			miR-128a	Antiproliferation Senescence	[85,90]
			miR-135a	Reduces tumorigenicity	[82,85,101]
			miR-218	Antiproliferation. Reduces clonogenicity Promotes differentiation	[102,103,104]
			miR-219	Antiproliferation Invasion Migration [85,104,105] (Ferretti et al., 2009, Genovesi et al., 2011, Shi et al., 2014)	[64,101,102]
			miR-324-5p	Proliferation	[82]
			miR-326	Reduces clonogenicity	[82]
**SUBGROUP 3**					
**miRNAs** **as Oncogenes**	**Cellular Function**	**Ref.**	**miRNAs** **as Suppressors**	**Cellular Function**	**Ref.**
			miR-204	IGF2R and LC3B target Anchorage-indipendent growth Invasion.Autophagy	[106]
			miR-218	Antiproliferation. Reduces clonogenicity Promotes differentiation	[102,103,104]
			miR-495	Gfi1 target Cell growth inhibition	[107]
			miR-1253	Pro-apoptosis Antiproliferation	[72]
			miR-9	Antiproliferation Differentiation Pro-apoptosis	[85,92]
**SUBGROUP 4**					
**miRNAs** **as Oncogenes**	**Cellular Function**	**Ref.**	**miRNAs** **as Suppressors**	**Cellular Function**	**Ref.**
			miR-9	Antiproliferation Differentiation Pro-apoptosis	[85,92]
			miR-204	IGF2R and LC3B target Anchorage-independent growth Invasion. Autophagy	[106]
			miR-495	Gfi1 target Cell growth inhibition	[107]
			miR-1253	Pro-apoptosis Antiproliferation	[72]
**SUBGROUP NOT SPECIFIED**					
**miRNAs** **as Oncogenes**	**Cellular Function**	**Ref.**	**miRNAs** **as Suppressors**	**Cellular Function**	**Ref.**
miR-21	Metastasis	[108]	miR-31	Antiproliferation	[85,109,110]
miR-106a/363	Proliferation Apoptosis Angiogenesis	[111]	miR-124	Differentiation Antiproliferation Pro-apoptosis	[82,112,113]
miR-106b	PTEN target Migration Invasion Tumor-sphere formation	[114]	miR-125a	Antiproliferation	[85]
miR-367	Invasion Proliferation	[115]	miR-199b-5p	Antiproliferation Reduces cancer stem cells	[91,116]
			miR-206	Antiproliferation	[58,93,117,118]
			miR-378	Differentiation Cell growth inhibition	[119]
			miR-383	Pro-apoptosis	[120,121]
